# Physical activity in people with dementia attending farm-based dementia day care – a comparative actigraphy study

**DOI:** 10.1186/s12877-020-01618-4

**Published:** 2020-06-22

**Authors:** B. Finnanger Garshol, L. H. Ellingsen-Dalskau, I. Pedersen

**Affiliations:** Department of Public Health Science, Faculty of Landscape and Society, Norwegian University of Life Sciences, Postbox 5003 NMBU, 1432 Ås, Norway

## Abstract

**Background:**

Despite public focus on the importance of physical activity and findings showing the benefits of such activity, research has shown that people with dementia are less physically active and have more sedentary behaviour compared to others in similar age groups. In Norway, there is a focus on day care services as a means to allow people with dementia to experience social, physical and cultural activities. Farm based services have been highlighted as an innovative and customized day care service, but little research has been done on physical activity and such services. This study therefor aims to investigate the potential of farm-based day care services as services that can promote physical activity for people with dementia.

**Methods:**

Actigraphy data from people with dementia attending farm-based day care services (*n* = 29) and people with dementia attending regular day care services (*n* = 107) was used to assess levels of physical activity in each group and to compare the two groups.

**Results:**

People attending farm-based day care had significantly higher levels of moderate activity, approximately 23 min each day, compared with persons attending ordinary day care (*p* = 0.048). Time spent in sedentary or light activity were similar for both groups. For the group attending farm-based day care services, days at the service, were significantly associated with less time spent in sedentary activity (*p* = 0.012) and more time spent in light (*p* < 0.001) and moderate activity (*p* = 0.032), and in taking more steps (*p* = 0.005) compared to days not at the service.

**Conclusion:**

The findings indicate that participants in farm-based day care for people with dementia have higher levels of physical activity compared to ordinary day care and that farm-based day care increases levels of physical activity for its attendees. Farm based day care services has the potential to help their participants reach or maintain recommended levels of physical activity. Further research is needed to investigate what facilitates this increase in activity and how such knowledge could be used in all types of day care services.

## Background

Physical activity and exercise can have many positive effects on people with dementia. It can improve physical functioning and basic activities of daily living [1, 2]; it can have a positive effect on cognitive function [3], and it can reduce levels of depression [4]. Additionally, both international and national guidelines highlight the importance of physical activity for older adults as a means to improve cardiorespiratory and muscular fitness, and functional health [5, 6]. Despite this, studies have found that people with dementia are less physically active, more sedentary and are more susceptible to physical decline than others in similar age groups [7–11], suggesting a need to promote physical activity among people with dementia.

Day care services for people with dementia are considered a setting that can help maintain physical function and provide opportunities for physical activity [12]. Day care services for people with dementia can be defined as adapted services, which aim to provide people with dementia the opportunity to experience social, physical and cultural experiences, and provide respite for caregivers [12]. In Norway, most day care services for people with dementia are located in conjunction with already existing institutions in the municipality (e.g. long-term care facilities, retirement homes) [13] and can be termed as regular day care services.

There is a call to innovate and create new services for people with dementia in Norway, and the Dementia Plan 2020 highlights farm-based dementia day care (FDC) as an example of varied and customized day care [12]. FDCs are structured similarly to regular day care, but base their activities on the farms resources and natural surroundings [14]. The farm as a care environment for people with dementia has been studied previously. Care farming can be defined as the use of commercial farms and agricultural landscapes as a base for promoting mental and physical health through normal farming activities [15]. Studies have observed that the participants at FDCs spend a large part of the day outdoors [14] and they are more actively involved in daily activities [16]. FDCs also stimulate dietary intake [17] and social participation [18], provide physical activity and contact with nature and animals [19], and the farm context enables activities and collaboration between participants and staff [20]. In addition, de Boer, Hamers, et al. [21] found in a study that people with dementia living in farm-based nursing homes had higher quality of life compared with residents of regular nursing homes.

There has been some research on day care services and physical activity. For regular day care services, van Alphen, Volkers, et al. [10] found that people with dementia attending these services were more active and less sedentary than people with dementia living in nursing homes, but less active and more sedentary than people without dementia living at home. Olsen, Pedersen, et al. [22] found similar results when comparing activity levels of people with dementia at regular day care with people with dementia in nursing homes. Strandenæs, Lund, et al. [23] found that participants reported that they felt attending regular day care helped them maintain physical functioning, and that it gave them opportunities for physical activity. At the same time, Strandenæs, Lund, et al. [24] found that while staff at regular day care centres highlighted the importance of physical activity, they tended not to offer specific training to strengthen the attendees.

There is seemingly little research on farm-based dementia care and physical activities and only De Bruin, Oosting, et al. [25] and de Boer, Hamers, et al. [26] seems to have investigated it. De Bruin, Oosting, et al. [25] found in an observational study that at FDCs activities (e.g. walking, crafts, watching animals etc.) were more frequent, more often outdoors, more aimed at individuals, and were of higher physical intensity than activities at regular day care facilities.de Boer, Hamers, et al. [26] found in their observational study of people with dementia living in different types of nursing homes, that participants living in farm-based nursing homes were more outside and more physically active. FDCs therefore seem to be an alternative day care service that could provide better opportunities for physical activity for people with dementia. However, little is still known about the level of physical activity at such services, and the difference between regular day care and FDC.

The present study therefore aims to investigate the potential of FDCs as services that can promote physical activity for people with dementia by comparing the levels of physical activity between attendees of regular day care and attendees of FDCs. In addition, it will compare levels of physical activity for people attending farm-based day care for the days they are at the farm and the days they are not. This may give a better understanding of what FDC can offer in relation to opportunities for physical activity. Based on existing research we expect to find that participants at FDCs have higher levels of physical activity than participants at regular day care services.

## Methods

The data used in the analyses was collected in two separate studies. The first study was a longitudinal study, and we used data collected at the second data collection point 6 months after baseline. We collected activity data, demographic data and information about degree of dementia and degree of physical functioning from participants attending FDCs (Study 1, [27]). The second study was a cross-sectional study by Olsen, Pedersen, et al. [22] (Study 2). From Study 2 we got similar data as in the first study, but this was collected form participants attending regular day care services for people with dementia.

### Participants and recruitment

**Fig. 1:**
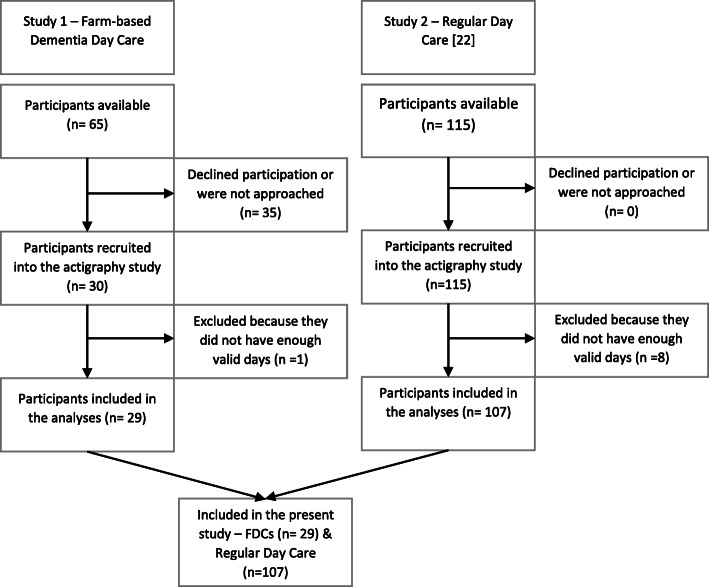
Flowchart of the inclusion of participants – The Flow chart shows the process of including participants from Study 1 and Study 2 in the analyses of the present study

In study 1, the municipality and the FDC were asked if they wanted to participate in the project. The day care service provider or a nurse in the municipality then conducted recruitment. Inclusion criteria for people with dementia were having attended a FDC for more than 3 weeks and seeing the same next of kin at least once a week. Age was not an inclusion criterion. In study 1, we recruited participant from late 2017 to late 2018. A total of 30 participants were recruited from 15 FDC services located all across Norway. In study 2, the development centres for dementia in three counties enrolled municipal day care centres. Data collection was conducted from early 2013 to mid-2014, and the staff at the enrolled centres conducted the recruitment of participants [22]. The inclusion criteria were 65 years or older and the person had to have either a dementia diagnosis or a score of < 25 on the Mini-Mental State Examination-test. A total of 115 participants from 23 day-care centres in the south-eastern part of Norway were included [22]. Figure 1 shows the inclusion of the participants from the two studies into the present study.

### Measurements

Both studies collected the following demographic data: age, gender, level of education, and level of dementia using a questionnaire. In Study 1, participants filled in the questionnaire together with a person from the research team, while in Study 2 the participants filled in the questionnaire together with staff at the day care centre. In addition, both studies used a test of physical functioning to assess functional mobility and an actigraph to measure levels of physical activity.

#### Level of physical activity

We used actigraphs (Actisleep+, Actigraph, Pensacola, US) to measure the level of physical activity. Actisleep+ is a 3-axis accelerometer approximately the size of a wristwatch. It measures physical activity levels, light exposure and sleep patterns. Actisleep+ measures movement along three axes: Vertically (Up and down), laterally (side to side) and longitudinally (forward and backward). It also measures the frequency and intensity/force of these movements. Using software, this is translated into measures of physical activity. The Actisleep+ does not register type of activities, nor their location. Actigraphy is a validated method for monitoring sleep and activity levels in people with dementia [28] and Erickson, Barr, et al. [29] demonstrated the feasibility of using actigraphy to measure physical activity in people with dementia. Additionally, several studies have used actigraphy to study levels of physical activity in people with dementia [10, 22]. The researchers introduced the actigraph orally, visually and in written form in both studies, both to the person with dementia and to their caregivers/relatives. In both studies, the participants wore the actigraph on the left wrist continuously for 7 days, these days included both days while at the day care services and days while not at the day care services. The participants could remove the actigraph, but were encouraged not to do so. Caregivers and relatives were also instructed to encourage the participants to put it back on if it had been removed by mistake.

#### Level of dementia

Both studies used Clinical Dementia Rating (CDR) scale to assess level of dementia. The scale comprises six items: memory, orientation, judgment and problem solving, community affairs, home and hobbies, and personal care. Each item is scored on a five-point scale from 0 to 3. 0 is considered normal, 0,5 very mild dementia, 1 is mild dementia, 2 is moderate dementia, while 3 is considered severe dementia. One overall score, following the same scale, is set based on the six items, giving precedence to memory [30]. CDR is considered a valid substitute for a dementia assessment when rating dementia and the severity of it [31, 32].

#### Level of functional mobility

Both studies used the Timed Up and Go-test (TUG) [33] to assess functional mobility, as we consider this to impact levels of physical activity. The timed Up and Go-test is a physical test were the participant rises from a chair, walks three meters, turns, walks back and sits down, while the test-administrator takes the time. In both studies, the TUG was administered according to Botolfsen and Helbostad [34], i.e. the testers repeated the test up to two times and the final score was the mean of the time, in seconds, for the two attempts.

### Statistical analysis

We processed the collected actigraphy data using the ActiLife-software, version 6.13.3 (ActiGraph, Pensacola, USA). To measure wear-time we subjected the data to a wear-time-validation. Wear-time validation allows the researcher to identify, based on a given set of parameters, invalid data. In this case, invalid data are periods when a participant has not worn the actigraph. We based the validation on the Troiano (2007) algorithm and excluded non-wear time from the subsequent analyses. We also applied a time filter between 08:00 and 20:00 to focus on day activity, as this is the timeframe where we believe the participants are the most active, and the timeframe in which day care centres could have an impact on the level of physical activity. We included days with more than 8 h recorded activity within that period as valid days. We decided that the participants would have to have at least three valid days to be included in the analyses, which is in accordance with findings from Hart, Swartz, et al. [35]. Of the 30 participants from study 1, only one participant was excluded from the analyses because of too few valid days, while in study 2, 8 participants were excluded because of too few valid days.

**Fig. 2:**
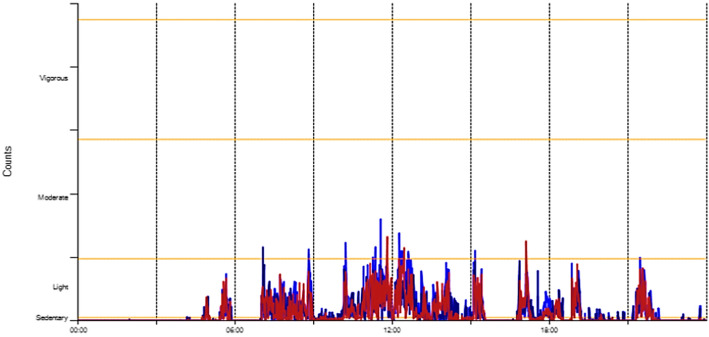
A representation of the actigraphy data – 24 h for one participant. The graph shows activity levels (y-axis) throughout the day (24 h) for one participant. The different colours represent different axes of movement

We processed the data further via the Scoring functions of the ActiLife-software. We calculated physical activity levels using the Freedson Adult Cut Points [36] in the ActiLife Software. ActiLife calculates activity levels based on the frequency and intensity of the registered movements. These constitute the measure counts and are specified as counts per minute (cpm). ActiLife categorises activity into five levels: sedentary (0–99 cpm), light (100–1951 cpm), moderate (1952–5742 cpm), vigorous (5743–9498), and very vigorous (> 9498). Sedentary activity is for example sitting and watching TV or sitting and listening to a conversation; light activity is for example standing or household activities, while moderate activity is for example walking. The Actigraph recorded the time spent on the different activity levels in minutes. Figure 2 shows a 24-h activity graph for one of the participants at FDCs. The graph shows when and for how long the participant was at the different activity levels. Through processing, Actilife subsequently expressed these as a percentage of the overall monitoring time. ActiLife also converts the data for a given time period into steps taken, giving us an estimate for each day for each participant. For the data from regular day care, only the percentages of activity levels were available for analysis.

We performed all statistical analyses using SPSS Statistics 25.0 (IBM Corp, Armonk, NY US) and we set the level of significance at 0.05. We used descriptive statistics to describe the two groups and an independent samples t-test to investigate differences between them. We used linear regression to further investigate the associations between type of day care and levels of physical activity, using data from both studies. We used the different levels of physical activity, based on the mean score for each participant, as the dependent variable and included type of day care service as an independent variable. Additionally, we included covariates that, based on previous research and existing differences between the two groups, could be confounders. Lastly, we used mixed model to investigate the difference in activity levels within group between days at the FDC and days without FDC, using only data from Study 1. In the linear mixed models, we used the levels of physical activity and steps taken each day as dependent variables, while attendance or non-attendance at the farm was included as an independent variable. As with the linear regression, we also included covariates that we considered as potential confounders. For both the linear regression analysis and mixed model analysis we analysed each of the levels of physical activity separately and built several models. Covariates were discarded from the models if they were not significant or did not significantly contribute to the model, for example as measured by r or Akaike information criterion (AIC). CDR and Living Alone were not included in the final analyses as they were not significant, and they did not contribute significantly to the model. In addition, despite there being a difference between the two groups on CDR, the group means were both in the “mild dementia”-category. The final models are presented in the tables in this paper.

### Ethics

Study 1 was approved by the Norwegian Centre for Research Data (NSD). Study 2 was approved by the Regional Committees for Medical and Health Research Ethics (REK). For the present study, we submitted an application to REK for sharing of data from study 2 to Study 1. This was approved on the basis that the data was anonymized.

All participants gave informed written consent and were informed that they could at any time withdraw from the studies. Since the Actisleep+ did not register type of activity and location, we considered it a non-invasive measure.

## Results

We present demographic data for the two groups and differences between the groups in Table 1. There were significantly more men among the participants attending FDC and they were significantly younger than those attending regular day care. In addition, among the participants attending FDC, fewer lived alone, more of them took prescription medication, and they had better TUG-mean time. The t-tests showed no group differences in time spent at different activity levels.

**Table 1:** Demographic data for participants attending farm-based dementia care or regular day care

Demographic	Farm-based dementia care (*n* = 29)	Regular Day Care (*n* = 107)	*P*-value^1^
Gender – man (%)	20 (69.0)	36 (34.0)	0.001
Age – mean (SD)	74.0 (7.22)	84.3 (8.10)	< 0.001
Education	*n* = 29	*n* = 87	0.189
- Primary (%)	4 (13.8)	40 (46.0)	
- Secondary (%)	19 (65.5)	20 (23.0)	
- College/University	6 (20.7)	25 (28.7)	
Living alone (%)	4 (13.8)	57 (54.3)	< 0.001
Clinical dementia rating (CDR)	*n* = 29	*N* = 102	
CDR – mean (SD)	1.22 (0.57)	1.52 (0.67)	0.021
CDR – groups			
- No (%)	0 (0)	4 (3.9)	
- Very mild (%)	3 (10.3)	3 (2.9)	
- Mild (%)	19 (65.5)	41 (40.2)	
- Moderate (%)	6 (20.7)	49 (48.0)	
- Severe (%)	1 (3.4)	5 (4.9)	
TUG – mean time in seconds (SD)	13.48 (5.61)	17.22 (8.02)	0.026
Activity levels			
Sedentary activity % - mean (SD)	39.70 (13.41)	43.51 (14.62)	0.209
Min – max^2^	11.75–66.19	10.67–73.41	
Light activity % - mean (SD)	50.53 (8.87)	50.19 (11.48)	0.864
Min-Max^2^	32.90–65.66	24.38–76.86	
Moderate activity % - mean (SD)	9.75 (9.34)	6.29 (5.97)	0,066
Min-Max^2^	0.17–38.43	0.03–28.35	

^1^*p* < 0.05 was considered significant

^2^Minimun and maximum values for the variable

Given the group differences on several demographic variables, we conducted an adjusted linear regression analysis (Table 2). This showed a statistically significant association between FDC and increased time spent at moderate physical activity. Participants attending FDCs spent 3.33% more time in moderate activity level each day than those attending regular day care. The average time registered for each day among the participants of FDCs were 685.18 min. This gives a 3.33% of 685.18 min, meaning the participants spent 22.81 min more in moderate activity each day, amounting to 159.67 min more for the entire week than those at regular day care. We found no such association for sedentary and light activity levels (data not shown).

**Table 2:** Linear regression for the association between type of day care, gender, age and TUG-sum on percentage of the time spent in moderate activity^1^

Variable	B (SE)	Beta	*p*-value^2^
Farm-based day care	3.33 (1.66)	0.20	0.048
Gender^3^	−5.01 (1.28)	−0.35	< 0.001
Age	−0.148 (0.08)	−0.18	0.073
TUG	−0.228 (0.079)	−0.24	0.005

^1^*r*^*2*^ = 0.250

^2^*p* < 0.05 was considered significant

^3^Women = 0, men = 1

To investigate within-group differences among participants attending FDC we ran linear mixed models (Table 3). When comparing days attending FDC with days not attending FDC, we found that days spent at the FDC were statistically significant associated with less time spent in sedentary activity, more time spent in light and medium activity and walking more steps. On the days at the farm the participants spent 25.85 min less in sedentary activity (*p* = 0.012), 40.37 min more in light activity (*p* < 0.001), 12.53 min more in moderate activity (*p* = 0.032) and took 1043.36 more steps (*p* = 0.005).

**Table 3:** Linear mixed models for the difference between days attending FDC and days not attending FDC with differing levels of physical activity or steps taken as dependent variable

Minutes in sedentary activity			
Variable	Estimate of fixed effects	95% CI	*p*-value^1^
Farm-based^2^	−25.84	(−45.81, − 5.88)	0.012
Gender	27.13	(−33.82, 88.09)	0.37
Age	−1.88	(−6.44, 2.66)	0.40
TUG	4.40	(−1.47, 10.28)	0.13
Minutes in light activity			
Farm-based	40.37	(24.69, 56.05)	< 0.001
Gender	36.96	(−11.85, 85.78)	0.132
Age	3.95	(0.30, 7.59)	0.035
TUG	−1.24	(−5.95, 3.46)	0.593
Minutes in medium activity			
Farm-based	12.53	(1.12, 23.95)	0.032
Gender	−44.85	(− 83.21, −6.48)	0.023
Age	−1.21	(−4.18, 1.55)	0.357
TUG	−3.11	(−6.81, 0.58)	0.096
Number of steps per day			
Farm-based	1043.36	(327.03, 1759.68)	0.005
Gender	− 467.62	(− 2771.26, 1836.02)	0.682
Age	−27.47	(−199.59, 144.64)	0.747
TUG	−38.59	(− 260.64, 183.45)	0.726

^1^*p* < 0.05 was considered significant

^2^Days on the farm = 1, days not on the farm = 0

## Discussion

The aim of the present study was to investigate the association between FDCs and activity levels in order to shed light on their potential as environments for promoting physical activity in the target group for the services. We wanted to investigate this both in relation to regular day care and for the attendees compared with their everyday life.

As our results show, attending FDCs is significantly associated with more physical activity, and at higher levels than attending regular day care. For the group attending FDCs, days spent on the farms were significantly associated with less sedentary activity, more light and moderate activity, and with more steps taken compared to days not at the farm. This is in line with previous research on farms as care settings for people with dementia [25, 26].

The higher levels in physical activity at the FDCs compared with regular day care could potentially be explained by several factors. The same factors could also potentially explain why people attending FDC have higher levels of physical activity on days at the farm compared to days not at the farm. One factor could be that the farm setting, to a larger degree, invites to physical activity through supplying the space for such activity and by having tasks that necessitates physical activity (e.g. woodcutting, gardening, feeding animals). Ibsen, Eriksen, et al. [14] noted that, while organized similarly to regular day care, FDCs in Norway differed in type of care environment with a wide range of activities and available resources. This included activities such as working with plants, tending and harvesting crops, woodworking and animal-related activities. Further, they found that the service took place in several areas, both on and outside of the farms such as the yard, the barn, gardens, a greenhouse and the surrounding uncultivated areas like forests and trails [14]. De Bruin, Oosting, et al. [25] also noted on the difference in activities between regular day care and FDCs. They found that activities at the FDCs were more often outdoors or in another building than regular day care. Additionally, activities at regular day care more often involved sitting, while activities at the FDCs more often involved standing or walking. de Boer, Hamers, et al. [26] observed similar results, but then in farm-based nursing homes. They found that the residents of farm-based nursing homes were more physically active, spent less time in passive activities, and were more engaged in their activities. Sudmann and Børsheim [19] found that the participants perceived the tasks at the FDCs as useful and meaningful, which could potentially increase their engagement in the task and the intensity. Further, Hassink, De Bruin, et al. [37] found that working with animals at care farms implicitly stimulated to physical activity. Lastly, de Bruin, de Boer, et al. [38] note that at FDCs activities are naturally incorporated into the environment and care provisions and are as such continuously present. Based on this previous research, activities at the FDCs, and especially the farm activities, seem to encourage higher levels of physical activity, than activities found at regular day care. The activities at, and inherent to, the FDCs can as such explain the higher levels of physical activity we found in our analyses. One avenue for future research could be to investigate if and how aspects of the farm setting could be transferred to other care settings for people with dementia.

Another factor explaining our findings could be the importance of the service providers as they are generally the ones who structure the day, and it is in many ways up to them how much focus there are on physical activity. While this is true for all types of day care services, the farmer has the added benefit of the farm resources and surroundings, and the knowhow that allows for their inclusion in the service. Sudmann and Børsheim [19] highlights the importance of the service provider as a facilitator for activities for the participants of FDCs, noting their roles as “work leader” and “host”. Within care farming in general the importance of the service provider has also been noted. Hassink, Elings, et al. [39] found that the personal and involved attitude of the farmer was considered a defining characteristic of care farms in general, and this is echoed in Steigen, Kogstad, et al. [40] which highlights the farmer as a significant important other to the participants. Pedersen, Ihlebaek, et al. [41] found that the participants, here people with clinical depression, reported that the farmers gave them tasks they could accomplish, leading to increased self-confidence and independence. Ellingsen-Dalskau, Morken, et al. [42] also note the positive effect of the involved farmer. In their study, the participants of a farm-based prevocational program reported that the farmers provided guidance, positive feedback and encouraged them to try new activities. The service provider’s engagement at the FDCs might facilitate increased physical activity through support, encouragement and the creating opportunities for the participants to experience coping. Low self-efficacy for going outdoors have for example been linked with restricting activities [43], and support from the service-provider could potentially alleviate this. Additionally, the service providers at farms could use their knowledge to facilitate and tailor activities more to the individual. De Bruin, Oosting, et al. [25] noted that activities at the FDCs were more often aimed at the individuals than at regular day care services, and that the regular day cares often had activities that included the entire group. Individualized activities have been noted as a facilitator for physical activity for people with dementia [44]. While there is a focus on tailoring activities to the individual at regular day care services, Strandenæs, Lund, et al. [24] found that staff at regular day care would state that they gathered individual knowledge about the attendees and tried to offer individualized services. At the same time, observations showed that the staff seemed to have insufficient knowledge about how to translate the information on the individuals into individually tailored and structured meaningful activities for the attendees. Additionally, the study found that there was a potential to include the attendees more in ongoing activities. This is mirrored in Myren, Enmarker, et al. [20] which found that participants at an FDC were more included in the daily activities at the FDC, like preparing meals, while participants at the regular day care centre were more passive in the daily activities. Therefore, the reason why we see differences, both between types of day care services, and days on and off the FDCs might be because the service providers promote physical activity both through providing organized activities to promote physical activity, such as taking walks or labour-intensive tasks, but also through support guidance, and individualization so that the participants try out farm activities which they might enjoy and exert themselves.

Both WHOs “Global Recommendations on Physical Activity for Health” [5] and Norwegian National Guidelines for physical activity for older people [45] gives recommendations on how much physical activity is necessary to maintain physical function and health. Additionally, the Norwegian guidelines recommend regular walks in varying terrain to maintain balance, range of motion and walking ability. Our findings indicate that attending FDCs could facilitate following these recommendations more so than attending regular day care. Further, our findings indicate that for those attending FDCs, the days on the farm are significantly more active and less sedentary. Given the high amounts of sedentary behaviour among people with dementia reported in previous research [7–10], the increased levels of physical activity on days with FDCs would seemingly be a valuable contribution towards less sedentary behaviour. That the participants are less sedentary are also in line with the WHO recommendations, as they highlight the need to avoid physical inactivity, as this has been identified as the fourth leading risk factor for global mortality [5]. Breaking up sedentary behaviour can also be important to maintain physical function in elderly people as Fujita, Fujiwara, et al. [46] and Shimada, Ishizaki, et al. [43] found when looking at the association between frequency of leaving the home and instrumental and basic activities of daily living. On the other hand, de Bruin, Oosting, et al. [47] found no significant difference between regular day care and FDCs in maintaining functional performance. Still, Blankevoort, van Heuvelen, et al. [1] found that physical activity improved physical functioning and basic activities of daily living among people with dementia. Additionally, they noted that higher levels of physical activity seemingly led to higher impact on physical functioning and activities of daily living. Based on current recommendations and previous research, our findings indicate that FDCs can potentially facilitate adherence to the recommendations and improve physical functioning.

### Strengths and weaknesses

The data for the present study were taken from two different projects, both with several data collectors, meaning we cannot discount inter-rater discrepancies with some of the measurements. However, the CDR [33, 34] and TUG [36] are both validated instruments with clear instructions. Also, the main measurement, the actigraphy, was assessed using the same software in both projects and data included in analyses based on the same guidelines, minimizing any discrepancies.

Further, the participants were not randomly select from among the relevant population. Recruitment was conducted by intermediaries, service providers and staff, in both studies. They might have screened their participants based on other criteria than the inclusion criteria, such as social or health status. This means that we might have a sample who are not representable for the whole population, potentially limiting the generalisability of the findings. The group attending regular day care and the group attending FDCs differed across several variables, such as gender, age, level of dementia and physical functioning. Additionally, it could be that participants attending FDCs are more physically active or have higher physical functioning, than people attending regular day care. To account for some of these differences we included age, gender and assessment of physical functioning in the statistical models as covariates as we believed these could influence levels of physical activity.

Last, one drawback with actigraphy, is that it does not give any information on the types of activity being conducted. As such, we do not know exactly what types of activities they are doing at the different types of day care services and which ones that contribute to physical activity. This means that we cannot preclude that the activities that contribute to physical activity at the FDCs are not specifically farm-related. Additionally, the way the actigraph measures activity means some forms of activity might be physically demanding, but not show up as physical activity with higher intensity [48]. This might be applicable for both groups, but more so for the ones attending FDCs due to the nature of the activities at the farms, for example carrying fodder for farm animals. However, a strength of using actigraphy is that it gives objective data on the participants physical activity and has been shown to be feasible for people with dementia [29], as relying on self-reported data alone has been shown to be unreliable [49, 50]. Non-wear can also be a challenge with actigraphy, but we conducted wear-time analyses and included requirements for what constituted a valid day to be included in the further analyses (minimum 8 h recorded activity in a 12-h time span), effectively minimizing the potential impact of non-wear.

The present study is cross-sectional, as such we do not have data about the participants baseline activity levels before attending day care, nor about the progression over time. This means we cannot infer causality based on the present study, but the results from the linear mix-model supports the assumption that attending FDCs is a main contributor to the higher levels of physical activity among their participants.

## Conclusions

As hypothesized the results of the present study indicate that participants attending FDCs have higher levels of physical activity compared to regular day care services and that FDCs increases physical activity levels for their attendees. FDCs has the potential to help their participants to reach or maintain the recommended amounts of physical activity stipulated in international and national guidelines. Further, previous research has shown that higher levels of physical activity can lead to health benefits for the participants. It can also aid them in maintaining physical function, and thereby maintain their activities of daily living. Based on previous studies, the farm setting and the service provider as a support for the participants and facilitator of individually tailored activities could explain the higher levels of physical activity at FDCs. Further research is needed to investigate what facilitates this increase in activity and which aspects promote physical activity and how such knowledge could be transferred between and used in all types of day care services.

## Data Availability

The datasets generated and/or analyzed during the current study are not publicly available due to them still being used for analyses but are available from the corresponding author on reasonable request.

## Abbreviations

FDC: Farm-based dementia day care
CDR: Clinical dementia rating scale
TUG: The timed up and go-test
